# Spontaneous small bowel evisceration through an umbilical region incisional hernia with features suggestive of Richter-type involvement in an HIV-positive patient: a case report

**DOI:** 10.1097/RC9.0000000000000654

**Published:** 2026-07-10

**Authors:** Naeem Shaikh, Tyaag Desai, Tintswalo Mabasa, Maheshwar Naidoo

**Affiliations:** a Department of Surgery, Ngwelezana Hospital, Empangeni, South Africa; b Nelson R Mandela School of Medicine, University of KwaZulu-Natal, Durban, South Africa; c Department of Surgery, University of KwaZulu-Natal, Durban, South Africa

**Keywords:** case report, evisceration, hernia, HIV, incisional, Richter

## Abstract

**Introduction and importance:**

Spontaneous bowel evisceration through a ventral hernia is a rare surgical emergency. Most reported cases involve primary umbilical hernias in the setting of chronic ascites. In contrast, cases involving incisional hernias are uncommon and poorly described. Richter-type involvement in spontaneous evisceration is not well described in this context.

**Case presentation:**

A 41-year-old human immunodeficiency virus (HIV)-positive male with a history of multiple laparotomies for a sigmoid volvulus and intra-abdominal sepsis presented with an irreducible umbilical region incisional hernia, followed by spontaneous rupture and small bowel evisceration. Clinical assessment and imaging demonstrated no features of complete bowel obstruction, although inflammatory markers were elevated. At laparotomy, approximately 25 cm of small bowel had herniated through a 3 cm fascial defect. The eviscerated segment involved the anti-mesenteric border and appeared dusky, with features suggestive of Richter-type involvement. Segmental bowel resection with primary anastomosis and primary hernia repair without mesh were performed. The postoperative course was uneventful.

**Clinical discussion:**

Spontaneous bowel evisceration can occur in incisional hernias, although it is rarely reported. Prior laparotomies, sepsis, and immunocompromised states may contribute to abdominal wall failure and reduced skin integrity. Richter-type involvement may progress to ischemia without the classical features of obstruction.

**Conclusion:**

Spontaneous evisceration through an incisional hernia is a rare but life-threatening complication. Early recognition and urgent surgical intervention are essential. Consideration should be given to early elective repair of incisional hernias in high-risk patients to prevent complications.

## Introduction

Incisional hernias arise due to impaired fascial healing following laparotomy and are associated with compromised tissue integrity and vascularity. Additional factors, such as infection, repeated surgery, and immunocompromised states, may predispose to progressive abdominal wall failure^[^1^]^.

Clinical wound-healing literature suggests that healed wounds regain approximately 80% of their original tensile strength, rendering previously operated tissue vulnerable to progressive weakness^[^2^]^. Spontaneous rupture of abdominal wall hernias is an uncommon but life-threatening surgical emergency. Most reported cases of hernia rupture and evisceration involve patients with primary umbilical hernias, typically with chronic ascites and associated increased abdominal pressure^[^3^]^. In contrast, spontaneous evisceration through incisional hernias is rarely described. Delays in elective hernia repair, especially in resource-limited settings, may increase the risk of such complications.

HIGHLIGHTS

- Spontaneous bowel evisceration through an incisional hernia is extremely rare.
- Richter-type bowel compromise may carry a risk of ischemia and perforation.
- Immunocompromise and prior laparotomies may increase the risk of hernia rupture.
- Early elective repair of incisional hernias may prevent catastrophic complications.

We present a case of spontaneous small bowel evisceration through an umbilical region incisional hernia in a human immunodeficiency virus (HIV)-positive patient, with features suggestive of a Richter-type involvement and early ischemia. Bowel resection and anastomosis with hernia repair were performed primarily in keeping with recommendations for contaminated fields. This procedure was carried out at a tertiary hospital in a low-middle-income setting.

## Methods

This case report has been presented in accordance with the Surgical CAse REport guidelines^[^4^]^.

## Case report

An HIV-positive 41-year-old male presented to a low-middle-income setting tertiary-level hospital with a 3-day history of an irreducible abdominal wall hernia and a 1-day history of spontaneous rupture through the overlying skin with bowel evisceration.

He had a history of a Hartmann’s procedure performed 4 years prior for a sigmoid volvulus. Five days after the index laparotomy, he underwent a relook laparotomy for fascial dehiscence secondary to sepsis from rectal stump disruption and colostomy breakdown. The stump was revised, the colostomy refashioned, and the abdomen temporarily closed. A relook 48 hours later confirmed the resolution of sepsis, and the abdomen was definitively closed. He was diagnosed with HIV during this admission and initiated on a fixed-dose, once-daily combination antiretroviral (ART) drug therapy consisting of tenofovir disoproxil fumarate, lamivudine, and dolutegravir. His cluster of differentiation (CD4) count at the time was 323 cells/μL, with a viral load of 84 900 copies/mL. At the time, his body-mass index (BMI) was 20.4 kg/m², and he had an albumin level of 30 g/L. He had no additional comorbidities and was a non-smoker. He was a piece-job worker and was not involved in heavy lifting or construction work.

One year later, he underwent a Hartmann’s reversal with an uneventful recovery. Subsequently, 1 year later, he developed an umbilical region incisional hernia, which was managed conservatively while awaiting elective repair.

On current presentation, the hernia became irreducible over 3 days, followed by a spontaneous rupture of the overlying skin and evisceration of the small bowel. His vital signs were within normal limits. Laboratory investigations revealed a raised white cell count of 13.2 × 10^9^/L and an elevated C-reactive protein of 286 mg/L. Hemoglobin was 12 g/dL, with normal renal and liver function. Lactate was 1.4 mmol/L, and potassium was 3.4 mmol/L. He reported adherence to ART and demonstrated a viral load on this admission of 69 copies/mL, suggesting compliance with antiretroviral therapy. A CD4 count on the current admission was unavailable; however, an improved CD4 count value of 619 cells/μL was recorded 1 year prior to this current admission. He had a body mass index of 24.2 kg/m^2^ with an albumin level of 42 g/L, an improvement from the index presentation several years earlier.

**Figure 1.**
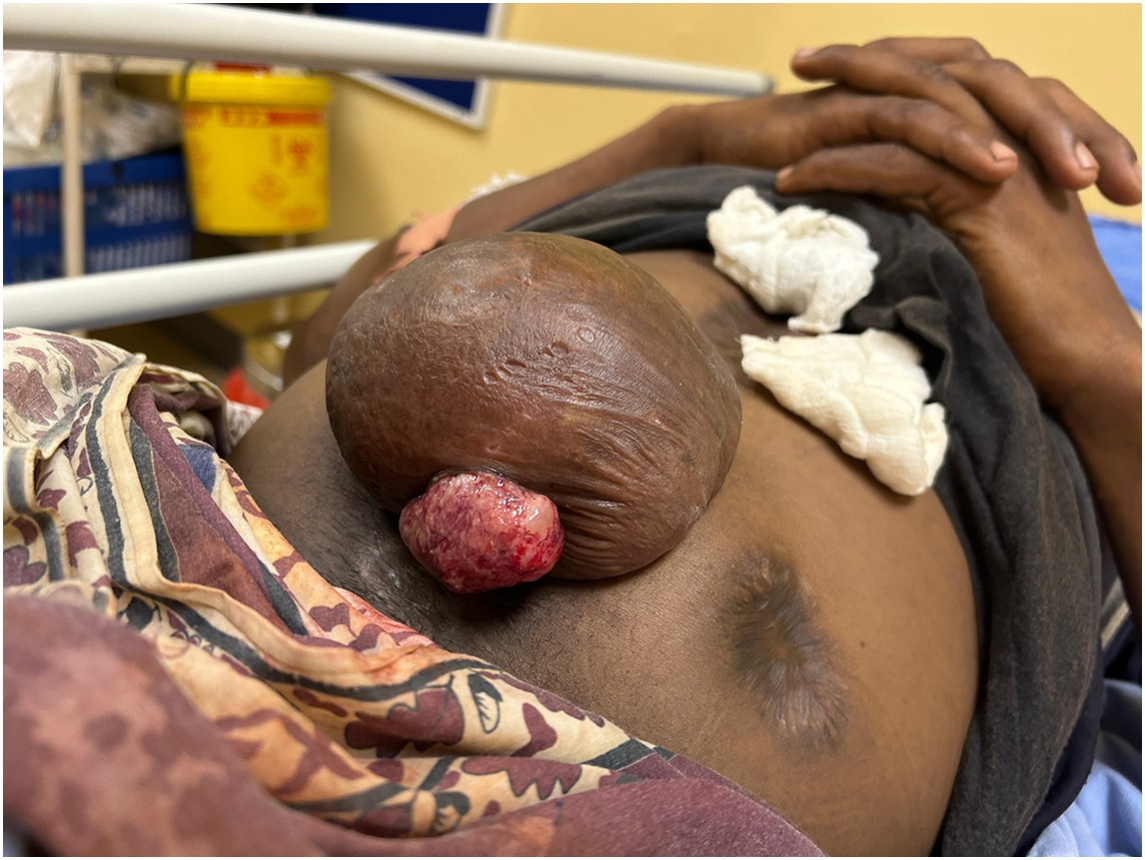
Clinical photograph demonstrating small bowel evisceration through a skin defect overlying an umbilical region incisional hernia.

Despite relatively normal biochemical parameters, the patient reported significant localized pain. Clinical examination revealed an enlarged, irreducible, and tender hernia with exposed, engorged, and discolored bowel (Fig. 1). Plain radiography showed no features of complete bowel obstruction. The exposed bowel was covered with paraffin-impregnated gauze to maintain moisture, and the patient was taken urgently to theater due to concern for bowel ischemia. He underwent surgery approximately 12 hours after presentation and arrived in theater within 2 hours of the decision to operate.

**Figure 2.**
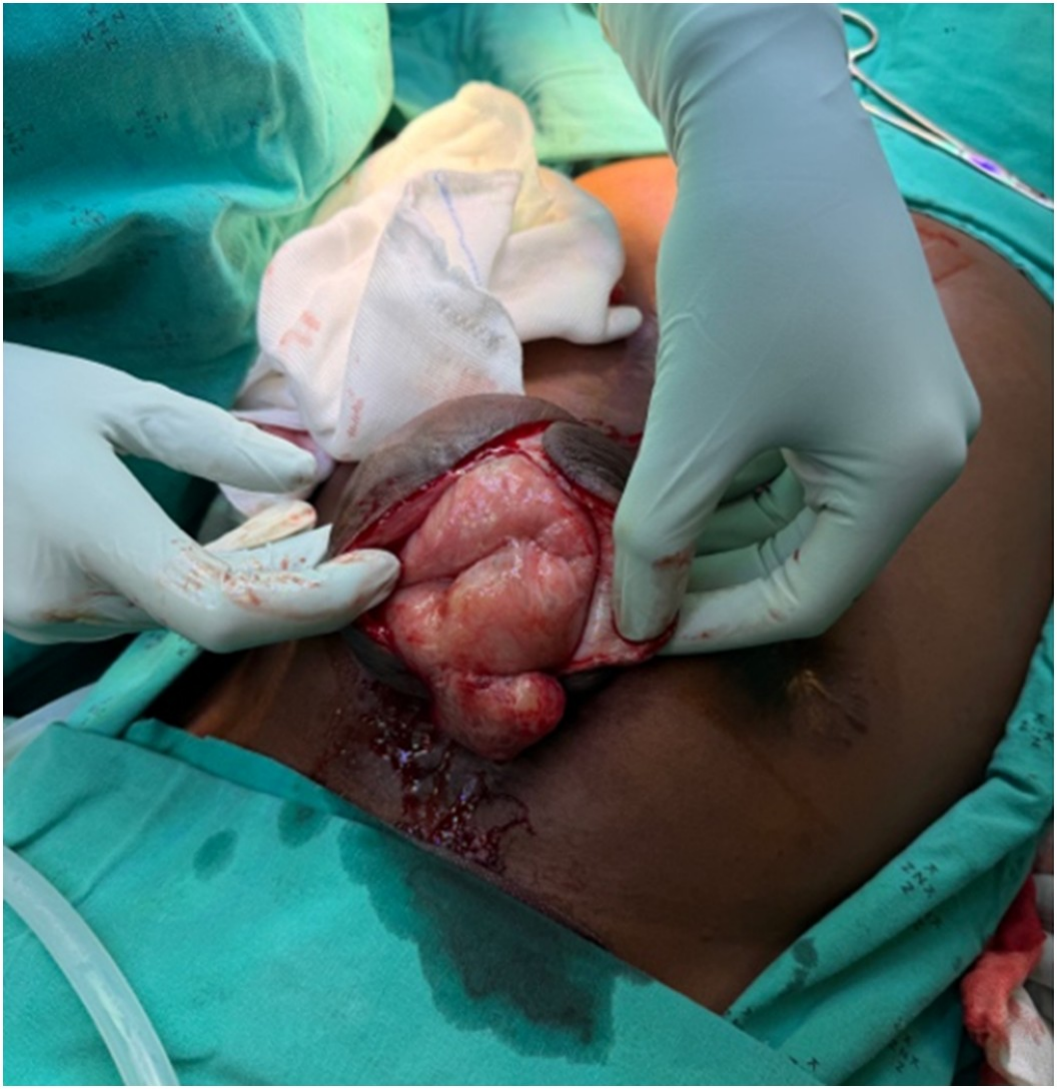
Entry into the hernia sac revealing distended bowel loops and approximately 25 cm of herniated small bowel with anti-mesenteric compromise, suggestive of Richter-type involvement.

**Figure 3.**
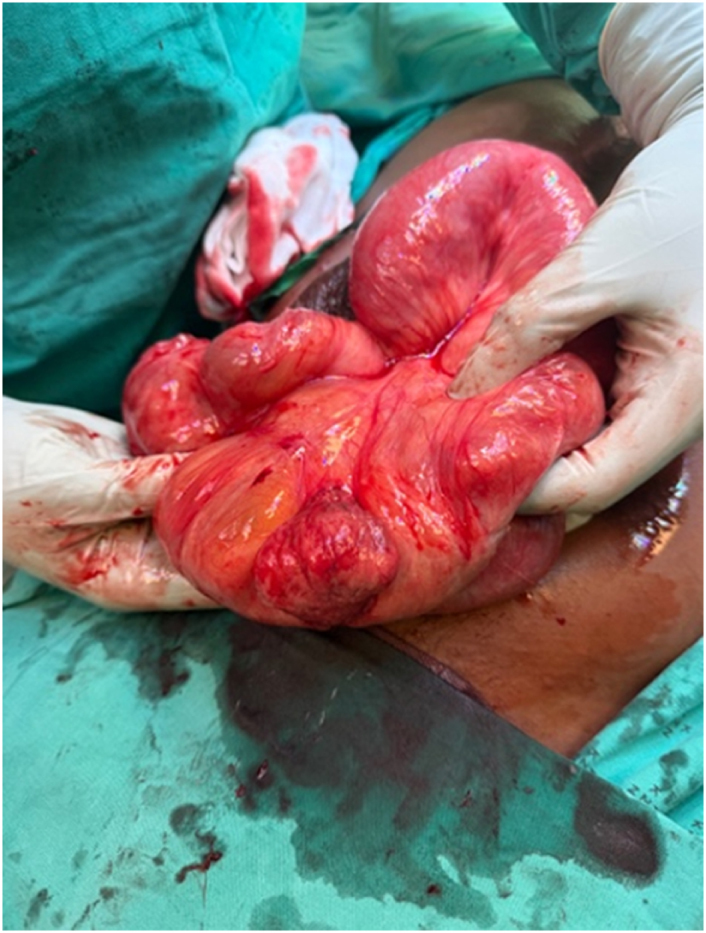
Intraoperative photograph demonstrating focal anti-mesenteric compromise of the eviscerated small bowel segment, with discolouration suggestive of early ischaemic change.

At laparotomy, the hernia sac was entered in layers via a 15-cm vertical midline incision. Approximately 25 cm of small bowel was found herniating through a 3 cm fascial defect (Fig. 2). The bowel was dilated, with soft interloop adhesions and fibrinous exudate. Approximately 200 mL of turbid intra-abdominal fluid was present in the pelvis, suggestive of early sepsis. The eviscerated segment involved a portion of the anti-mesenteric border of the small bowel, with features suggestive of Richter-type involvement, and appeared engorged and dusky (Fig. 3).

Meticulous blunt dissection was performed to free adhesions without inadvertent small bowel injury. Segmental small bowel resection was performed, followed by a hand-sewn end-to-end primary anastomosis using 3–0 Monocryl. The abdominal cavity was irrigated with normal saline before returning the bowel to the abdominal cavity.

The hernia sac was dissected from the overlying skin and excised. Due to the contaminated field and concern for sepsis, the fascial defect was repaired primarily without mesh, using interrupted polydioxanone sutures. Redundant skin was excised, and the wound was closed with interrupted non-absorbable sutures, incorporating healthy skin from beyond the borders of the previous incision scar. The total operative time was approximately 90 minutes, with total blood loss estimated at 150 mL.

The patient had an uneventful postoperative recovery. His pain improved significantly, and he remained clinically stable throughout admission. He received standard postoperative thromboprophylaxis with subcutaneous enoxaparin according to institutional protocol. He was tolerating a full ward diet and passing stool. His wound had healed, and after completing 7 days of intravenous amoxicillin–clavulanate, he was discharged and had his sutures removed on day 10 at the local clinic. Histopathology of the resected bowel segment demonstrated sections of small bowel with edema and congestion, with features consistent with ischemia with peritonitis. There were no granulomas, viral inclusions, or evidence of malignancy. A 5-week postoperative telephone consultation revealed no recurrence of the hernia.

## Discussion

Incisional hernias are defined as a protrusion of abdominal viscera through a weakened area in the abdominal wall following prior surgery. They occur in approximately 15–20% of laparotomies and may present months to years after surgery, most commonly within the first 3 years^[^5^]^. The pathogenesis of progressive abdominal wall weakness is multifactorial, involving both patient- and surgery-related factors. Patient-related risk factors that impair wound healing include smoking, obesity, increased abdominal pressure, wound infection, and chronic illnesses such as diabetes and poor nutrition. Surgical risk factors include inadequate suturing technique, poor tissue handling, inappropriate suture material, and excessive tension at closure.^[^5^]^

While incisional hernias are relatively common, spontaneous rupture with bowel evisceration is a rare surgical emergency. Evisceration is defined as the protrusion of internal viscera through an abdominal wall defect with exposure to the external environment. This significantly increases the complexity of management due to the risk of contamination and bowel compromise. In our case, the small bowel eviscerated through a weakened area in the skin with partial bowel involvement. The constricted portion posed a significant risk of ischemia and perforation. The overlying skin was likely compromised due to chronic tension and impaired vascularity.

Previous studies have demonstrated that healed skin achieves approximately 80% of the original tensile strength^[^2^]^, thereby highlighting the vulnerability of previously operated tissue. Repeated laparotomies and prior surgical site sepsis, particularly in cases that heal by secondary intention, compromise both fascial and cutaneous integrity, predisposing to hernia formation and eventual skin rupture.

Most reported cases of spontaneous hernia rupture involve primary umbilical hernias, typically in patients with chronic ascites or long-standing defects, as demonstrated in a recent review by Gianazza *et al*^[^3^]^. In contrast, spontaneous evisceration through incisional hernias remains rarely reported. A focused review was performed using PubMed and Google Scholar to identify previously reported cases of spontaneous evisceration through incisional hernias up to March 2026 (Table 1). Search items included “incisional hernia,” “spontaneous rupture,” and “evisceration.” Only adult cases were included. Cases involving inguinal hernias, primary ventral hernias, trauma, paediatric patients or ascites-related rupture were excluded.

**Table 1:** Previously reported cases of spontaneous evisceration through incisional hernias.

Author (year)	Age/sex	Hernia type	Risk factors	Involved bowel	Management	Outcome
Beltrán *et al* (2006)^[^6^]^	81/F	Infra-umbilical incisional	Prior surgery	Small bowel	Hernia polypropylene mesh repair.	Two-year follow-up free of complications
Eke (2006)^[^7^]^	28/F	Midline incisional hernia	Coughing bout. Two-month hernia history. Prior midline caesarean section. HIV positive.	Small bowel	Resection and anastomosis. Primary hernia repair.	One-month review no recurrence.
Gupta *et al* (2011)^[^8^]^	45/F	Lower midline incisional hernia.	Coughing bout. Five-year hernia history. Prior surgery for total hysterectomy and bilateral salpingo-oophorectomy complicated with wound dehiscence.	Small bowel	Primary hernia repair.	Asymptomatic 4-month follow-up
Martis *et al* (2011)^[^9^]^	60/F	Incisional	Coughing bout. Prior surgery for tubectomy. Thirty-year hernia history.	Small bowel	Hernia repair with prolene mesh.	Asymptomatic 3-month follow–up.
Das *et al* (2011)^[^10^]^	53/F	Umbilical incisional	Prior laparoscopic cholecystectomy. Two-year hernia history.	Inflamed appendix, part of caecum and terminal ileum	Appendectomy. Primary hernia repair.	Uneventful postoperative period.
Akkucuk *et al* (2013)^[^11^]^	65/F	Recurrent Pfannenstiel incisional hernia	Multiple surgeries for recurrent hernia.	Small bowel	Ileal resection and end-to-end ileo-ileal anastomosis. Hernia repair with mesh.	Six-month follow-up no recurrence.
Sailaja *et al* (2013)^[^12^]^	50/F	Midline incisional	Bending for prayer. Previous hysterectomy for endometrial carcinoma.	Small bowel	Resection and end-to-end anastomosis. Primary hernia repair.	Asymptomatic 2-month follow-up.
Agodirin *et al* (2015)^[^13^]^	34/F	Lower midline incisional	Heavy lifting. Two previous caesarean operations complicated with wound dehiscence. Four-month hernia history.	Small bowel	Primary hernia repair.	Twelve-month follow-up no recurrence.
Roy *et al* (2015)^[^14^]^	55/F	Infra-umbilical Incisional	Prior total abdominal hysterectomy. Five-year hernia	Small bowel	Primary repair of iatrogenic small bowel injury. Primary hernia repair	One-year follow-up no recurrence.
Osei-Tutu *et al* (2016)^[^15^]^	56/F	Incisional	Chronic cough. Prior abdominal surgery. Two-year hernia history. HIV positive.	Small bowel	Primary hernia repair	Lost to follow-up after discharge.
West *et al* (2016)^[^16^]^	70/F	Midline incisional	Prior open abdominal aortic aneurysm repair.	Appendix and greater omentum.	Appendectomy. Hernia repair with collagen-based mesh.	Seven-week follow-up no recurrence or infection.
Rambhajan *et al* (2016)^[^17^]^	80/F	Upper midline incisional	One-week history of constipation. Prior surgery for bilateral staghorn calculi. Thirteen-year hernia history.	Small bowel	Hernia repair with mesh.	Survived
Ansari *et al* (2017)^[^18^]^	40/F	Midline incisional	Eight-year hernia history. Prior laparotomy for ileal perforation	Small bowel	Hernia repair with mesh.	Two-year follow-up no recurrence.
Edeh *et al* (2018)^[^19^]^	56/F	Incisional	Coughing bout. Nine-month hernia history. Prior laparotomy for uterine leiomyoma.	Small bowel	Hernia repair with mesh.	Follow-up at 6 months no recurrence.
Thakkar *et al* (2019)^[^20^]^	35/F	Incisional	Heavy lifting. Previous laparotomy. Two-year hernia history.	Omentum	Resection of omentum. Hernia repair with mesh.	Survived
Weledji *et al* (2020)^[^21^]^	55/F	Lower midline incisional	Previous laparotomy for hysterectomy complicated with urinary fistula and second laparotomy for repair. More than 5-year hernia history. Pressure necrosis.	Small bowel	Resection and ileoileal anastomosis. Primary hernia repair.	Three-month follow-up no recurrence
Thalahitiyage *et al* (2021)^[^22^]^	62/F	Pfannenstiel Incisional hernia.	Prior curative hysterectomy for endometrial adenocarcinoma complicated by wound sepsis. Neglected hernia.	Small bowel	Hernia repair with mesh.	One-month follow-up asymptomatic.
Das *et al* (2022)^[^23^]^	48/F	Umbilical midline incisional	Prior laparotomy.	Small bowel	Primary hernia repair.	Survived
Bhagvat *et al* (2023)^[^24^]^	58/M	Incisional hernia	Coughing bout. Prior laparotomy for feeding jejunostomy for esophageal carcinoma.	Small bowel	Resection and double barrel ileostomy done. Primary hernia repair.	Survived
Shiva *et al* (2024)^[^25^]^	74/M	Incisional	Chronic smoker and cough. Straining while defecation. Previous laparotomy for gastric perforation Chronic hernia history.	Small bowel	Temporary abdominal closure.	Survived
Kumar *et al* (2025)^[^26^]^	40/F	Incisional	Heavy lifting. Prior laparotomy for perforation peritonitis complicated by fascial dehiscence. Chronic hernia history.	Small bowel	Hernia repair with mesh.	One-year follow-up no recurrence.

Twenty-one cases were identified, the majority involving small bowel evisceration, except for three involving the omentum or appendix^[^10,16,20^]^. Five cases required bowel resection due to compromised viability^[^7,11,12,21,24^]^ while several cases utilized mesh repair, including one utilizing a collagen-based mesh^[^16^]^. Four cases involved risk factors related to previous wound complications, including wound sepsis and dehiscence^[^8,13,22,26^]^. One case mentioned a history of repeated friction on the skin, leading to ulceration and eventual rupture^[^21^]^. HIV positivity has been rarely described among previously reported cases^[^7,15^]^.

These findings support the role of prior abdominal surgery and compromised abdominal wall integrity as key predisposing risk factors. Despite the severity, favorable outcomes were generally achieved with prompt operative management.

A notable feature of this case is the apparent Richter-type pattern of bowel compromise. A classical Richter’s hernia is characterized by the entrapment of only part of the bowel circumference, typically the anti-mesenteric border, at the level of the fascial defect, and may occur without complete bowel obstruction^[^27^]^. In this case, the bowel had already herniated through the fascial ring, while the anti-mesenteric portion appeared compromised at the level of the skin defect rather than solely at the fascial ring. Although this does not represent a classical Richter hernia, the operative and histopathological findings support features suggestive of a Richter-type mechanism. To our knowledge, we have not encountered a clearly documented previous case of spontaneous incisional hernia evisceration with partial anti-mesenteric bowel wall compromise resembling a Richter-type mechanism. Due to localized tightness, such involvement can progress to ischemia, necrosis, and perforation, necessitating the need for urgent intervention.

The patient’s HIV status may have further contributed to impaired wound healing and tissue integrity in combination with other risk factors. At index presentation, he was ART-naïve with a CD4 count of 323 cells/μL, hypoalbuminemia, intra-abdominal sepsis with fascial dehiscence, and required multiple operations. On current admission, he reported adherence to ART with a significant decrease in viral load to 69 copies/mL, an improved BMI of 24.2 kg/m^2^ and an albumin level of 42 g/L. His postoperative recovery was uneventful. While HIV infection has not been definitively established as an independent risk factor for incisional hernia development, it may exacerbate impaired wound healing with increased susceptibility to infection, particularly in the presence of sepsis and repeated surgical intervention, as seen in this case. Despite the paucity of data, available clinical literature suggests that immunosuppression may impair wound healing through delayed inflammatory regulation, altered collagen synthesis, and increased susceptibility to wound infection^[^28,29^]^. These findings support the importance of optimizing the immune system and nutritional status in patients at risk for impaired wound healing.

This case highlights the importance of early elective repair of incisional hernias, particularly in high-risk patients with multiple previous surgeries or immunocompromised states. Delayed management may result in complications such as spontaneous rupture and evisceration. Learning points include identifying methods to mitigate the development of incisional hernias, appropriate management of wounds to prevent surgical site sepsis, especially in immunocompromised states, and early recognition of bowel compromise in the setting of herniation or evisceration.

In conclusion, spontaneous evisceration through an incisional hernia is a rare but life-threatening complication. This case highlights a possible Richter-type mechanism and underscores the potential contribution of prior surgery, sepsis, and immunocompromise to progressive abdominal wall failure. Early recognition, prompt intervention, and appropriate operative decision-making are essential for favorable outcomes. Greater awareness of this rare complication may support earlier elective repair of incisional hernias in high-risk patients, potentially preventing catastrophic outcomes.

## Patient perspective

Following treatment, I noticed an improvement in my symptoms. I am grateful for the care and hope that sharing my experience may help others notice similar symptoms and seek early treatment. (Translated into English due to language difference)

## Strengths and limitations

Key strengths include detailed operative findings, histopathological correlation, focused comparison with previously reported cases of spontaneous evisceration through incisional hernias, and adherence to accepted surgical practices.

Limitations include the rarity of case design, short follow-up, and the absence of a CD4 count value during this admission.

## Ethical approval

The case report was conducted in accordance to local policy. Formal ethical approval was not required as this was a retrospective review of anonymised case data.

## Data Availability

Data sharing is not applicable to this article, as no datasets were generated or analyzed.

## Research registration unique identifying number (UIN)

Not required for this case report, as per institutional guidelines.

## Presentation

This case has not been presented at a conference or regional meeting.

## Artificial Intelligence declaration

Artificial Intelligence (AI; ChatGPT 5.5 Thinking, accessed May 2026) was used to assist with manuscript structuring and language editing. De-identified clinical text was used; no patient names, hospital numbers, dates of birth, or directly identifying information were entered. No facial or directly identifying clinical images were entered. All AI-generated content was critically reviewed, fact-checked, edited, and approved by the authors. The authors take full responsibility for the integrity and accuracy of the manuscript and declare no interests or financial relationships with the AI vendors.
